# Non O1 *Vibrio cholerae* as a cause of bacteremic lower limb cellulitis: A case report

**DOI:** 10.1016/j.ijscr.2019.09.020

**Published:** 2019-09-24

**Authors:** Tarig Ahmed Abdelhafiz, Amani Mansour Alnimr, Abdulrahman Mohammed Alabduljabbar, Hussain Salman AlMuqallad, Abdullah Abdulmonem Alzarra, Hassan Nasser Alrashed, Moudhi Mufarej Aladwani, Amani Mohammed Hakami

**Affiliations:** a Department of General Surgery, King Fahd Hospital of the University, Saudi Arabia; b Department of Microbiology, King Fahd Hospital of the University, Saudi Arabia; c King Fahd University Hospital, Imam Abdulrahman Bin Faisal University, Saudi Arabia; d College of Medicine, Imam Abdulrahman bin Faisal University, Dammam, Saudi Arabia

**Keywords:** Non-O1, non-O139 *Vibrio cholerae*, Cellulitis, Diabetic foot

## Abstract

**Introduction:**

Self-limiting gastroenteritis and wound infections in immunocompetent patients are most of the cases involved with Non-O1, non-O139 *Vibrio cholerae* which its microorganism occasionally accountable for intestinal and extra-intestinal infections. Cellulitis is an infection of the skin and underlying soft tissue mostly affected by bacteria. Diabetic foot ulcer is the most costly and devastating complication of diabetes mellitus.

**Case presentation:**

This following case describes a bacteremic soft tissue infection in a diabetic patient who is 54-year-old male with 6 years of type 2 diabetes mellitus. The patient was treated with surgical debridement of the foot ulcer, calf fasciotomy and medical therapy then he discharged. The next day readmitted with deteriorating of his condition the whole right leg to the level of the knee became ischemic and black with evidence of wet gangrene. A Guillotine above knee amputation was done.

**Discussion:**

Upon review of literature, we only found few cases have been reported of cellulitis due to *V. cholerae*. A degree of hepatic impairment or immunocompromised state like Diabetes Mellitus, chronic infections, malignancies, and peripheral vascular disease are seen in the majority of non-gastrointestinal *V. cholerae* infections which suggests that it should be included in the differential diagnosis of bacteremic skin and soft tissue infections in patients with underlying illnesses.

**Conclusion:**

Cholerae should be included in the differential diagnosis of any bacteremic skin and soft tissue infections especially in elderly, comorbid and immune-compromised patients. Well-timed, proper antibiotic and surgical treatments are important in management of the infection to decrease morbidity and mortality.

## Highlights

• Non-O1, non-O139 *Vibrio cholerae* is an uncommon cause of cellulitis.
• *Vibrio cholerae* should be included in the differential diagnosis of any bacteremic skin and soft tissue infections especially in elderly, comorbid and immune-compromised patients.
• The aim of this report is to review the literature and highlight how the diagnosis and management can be optimized.
• Well-timed, proper antibiotic and surgical treatments are important in the management of the infection to decrease morbidity and mortality.

## 1 Introduction

The work has been reported in line with the SCARE criteria [1]. Diabetic foot syndrome is one of the most significant and devastating complications of diabetes and its well-defined as a foot affected by ulceration that is associated with neuropathy and/or peripheral arterial disease of the lower extremities in a patient with diabetes [2]. The annual incidence of foot ulceration is estimated to be approximately 1%–4%, and its prevalence ranges from 4% to 10%, however, the lifetime risk for the development of a diabetic foot ulcer in patients with diabetes ranges from 15% to as high as 25% and presence of foot ulceration is considered to be the main precursor of a lower extremity amputation among patients with diabetes [2]. Although, polymicrobial infections including aerobic gram positive, negative, and anaerobic bacteria are very common in diabetic foot ulcers [3]. However, pathogens like *Vibrio* spp. could be infrequently isolated. These are Halophilic facultative anaerobic organisms. Hence, the presence of diabetic foot associated with infection by this organism, complicated by bacteremia is a rare condition since *V. cholerae* is more encountered in cases of acute gastroenteritis. To our knowledge, this is the first case report that describes the occurrence of bacteremic *V. cholerae* cellulitis in a diabetic foot in Saudi. Therefore, the aim of this report is to review the literature and highlight how the diagnosis and management can be optimized.

## 2 Case report

A 54 years old Saudi man, known case of Hypertension, Diabetes Miletus type II for 6 years with history of percutaneous coronary intervention which has been done 2016, due to Ischemic heart disease, atrial fibrillation and old stroke. He presented to emergency department with history of acute confessional state for 1-day duration. He was in his usual state of health till 1 days prior to admission when he presented with symptoms including fever, confusion and decrease oral intake associated with right leg pain. He was discharged recently from the hospital – 2 weeks back – when he was complaining of obvious swollen and erythematous on the right leg below the knee a diagnosis of Right lower limb cellulitis was established, he was vitally stable and treated by antibiotics (clindamycin, ciprofloxacin, meropenem). There was no history of limbs weakness, loss of consciences, seizure, trauma nor facial asymmetry. Furthermore, He denied a recent travel and ingestion of raw seafood. On physical examination, the patient was moderately obese, appeared very ill and agitated. Had temperature of 37.6 °C heart rate of 112 beats per min, respiratory rate of 20 breaths per min, blood pressure of 150/93 mm of mercury, and normal oxygen saturation while breathing room air as measured by pulse oximetry, Glasgow coma scale 14 out of 15. His lungs were clear, he was tachycardic, and the abdomen was normal without organomegaly. The right lower extremity showed gross swelling of the right lower limb up to the level of the thighs with tense right calf muscles, evident bullae and pus discharge from the sole of the right foot. Laboratory tests (Table 1) revealed a leukocytosis of 16.3 × 10^9^/L, elevation of C-Reactive Protein (15.30 mg/dL) and liver enzymes.

**Table 1:** Laboratory test results at admission and discharge.

	Normal range (units)	Admission 27/07/2018	Discharge 02/09/2018
Hemoglobin	13–18 (g/dL)	13.7	9.6
Leucocytes	4.0–11.0 (×10^9^/L)	16.3	10
Platelets	150–450 (×10^9^/L)	272	520
INR	–	1.29	2.72
aPTT	30.6 (s)	38	30.7
Glucose	70–110 (mg/dL)	117	133
BUN	7–18 (mg/dL)	35	–
Creatinine	0.8–1.3 (mg/dL)	1.81	–
Sodium	135–145 (mmol/L)	140	135
Potassium	3.6–5.2 (mmol/L)	4.40	4.40
Chloride	98–106 (mmol/L)	103	103
Total Bilirubin	0.3–1.2 (mg/dL)	1.1	–
Direct Bilirubin	<0.5 (mg/dL)	0.60	–
Alkaline Phosphatase	30–120 (UI/L)	188	–
Gamma-Glutamyl Transferase	<55 (UI/L)	141	
Aspartate Aminotransferase	8–35 (UI/L)	41	–
Alanine Aminotransferase	10–45 (UI/L)	9	–
C-Reactive Protein	0.01–0.82 (mg/dL)	15.30	–
TROPININ I	<34.2 (pg/mL)	20 PG/ML	–

**Fig. 1:**
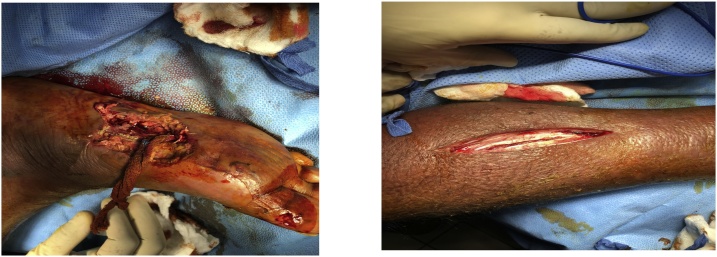
Surgical debridement of the right foot ulcer (right) with right calf fasciotomy (left).

The patient was diagnosed to have infected right foot and impending sepsis. Sepsis protocol was initiated including cultures of blood and wounds. He was admitted on 27/07/2018 to the surgical intensive care unit and underwent surgical debridement of the right foot ulcer with right calf fasciotomy on 28/07/2018 (Fig. 1).

He remained in the surgical intensive care unit for 16 days for further resuscitation. The initial blood culture taken on 27/07/2018 flagged positive for *Vibrio cholerae* after 8.5 h of incubation in the BACT/ALERT® VIRTUO® (bioMérieux, US). Microscopic examination showed short Gram negative, slightly curved rods (image). As per the lab protocol, it was subcultured on blood, chocolate and Macconkey agar plates. 18 h later, pure growth of non-lactose fermneters was noted which gave positive oxidase and catalase reactions. Vitek MS (matrix-assisted laser desorption ionization-time of flight MS) gave the identity as *V. cholerae*.

Manual testing confirmed it showing it as Non O1, Non O139 serotype. The organism was grown in TCBS Selective medium (SPML, Saudi Arabia) at 42 C as yellow colonies.

Biochemical reactions, nitrite negative, lysine negative, VP positive and ONPG positive) were illustrated using Vitek II (bioMérieux, US) using the VITEK®2 GN ID card. Non-halophilic nature of the spp. was shown by ability to grow on specialized low salt medium, CLED (SPML, Saudi Arabia).

**Fig. 2:**
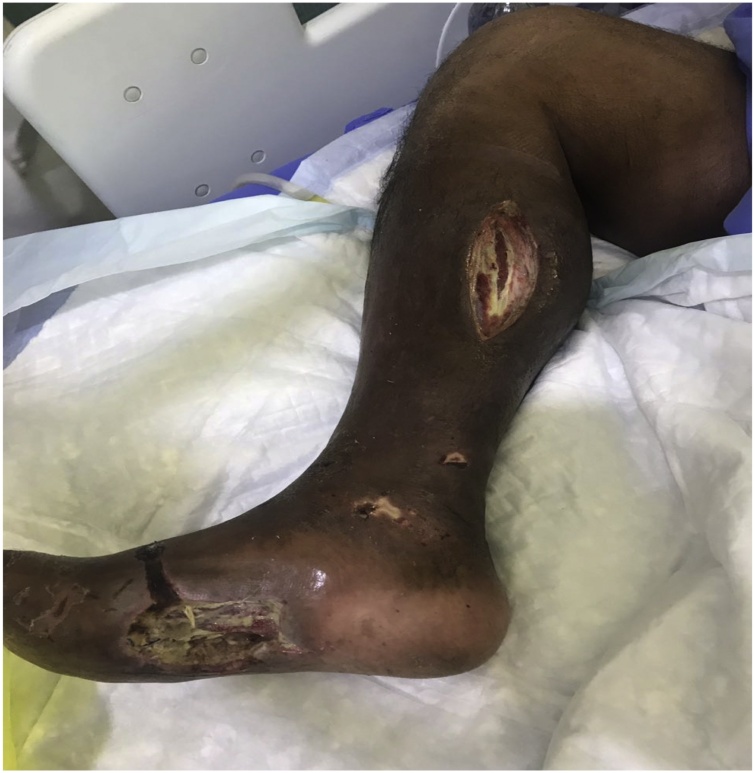
Right leg Ischemic and evidence of Gangrene.

The initial antimicrobial therapy was of 1 mg IVP of Meropenem, Vancomycin and Tigacyclin 50 mg IVP. The *Vibrio cholerae* showed antibiotic-resistant to ampicillin. He also received further medications during his Hospital stay. He was moved to surgical ward on 06/08/2018. The right foot wound has improved with healthy granulation tissue on the right leg wound, however, right foot still has some slough along granulation tissue. Therefore, the patient was vitally stable, ready for discharge on 02/09/2018 and to continue daily dressings as an out-patient. The next day 03/09/2018 patient readmitted with urosepsis to medical word. The Surgical team of the hospital followed up as consultation for the progress of the wound and dressings continued. The calf wound was very clean however, the foot wound has minimal slough that needed daily dressings and occasionally bed side debridement. On 03/10/2018 he intubated again due to overwhelming sepsis and set on inotropic support and transferred to medical ICU. Due to the vasopressor effects the whole right leg to the level of the knee became ischemic and black with evidence of wet gangrene (Fig. 2).

Guillotine above knee amputation was done on 07/10/2018 and then patient returned to the medical ICU and continued on inotropic support. Eventually, he passed away at 2:00 am on 09/10/2018.

## 3 Discussion

Non-O, 1 non-O139 *Vibrio cholerae* is an uncommon cause of cellulitis. There are no reports of such cases in diabetic foot infections. This case describes a bacteremic soft tissue infection in a diabetic patient who is Elderly with Chronic diseases. Diabetic foot ulcer is defined as a full-thickness wound which is present at a level distal to the ankle in patients with diabetes. The annual incidence of foot ulceration is estimated to be approximately 1%–4%, and its prevalence ranges from 4% to 10%, however, the lifetime risk for the development of a diabetic foot ulcer in patients with diabetes ranges from 15% to as high as 25% and presence of foot ulceration is considered to be the main precursor of a lower extremity amputation among patients with diabetes. The first step in ulcer prevention is the careful screening for foot problems and detection of patients at high risk. Effective management of the different sides of diabetic foot syndrome with Multi-disciplinary team approach is required. Standard wound care is recommended, while modern treatment modalities have shown some promising results in recent studies. Thereafter, diabetic foot ulceration is preventable with proper intervention [2].

In most cases of *Vibrio cholera*, usually acute gastroenteritis is the most common clinical symptom for both sporadic and outbreak cases. It is rarely involved in extra-intestinal infections, which include primary bacteremia, skin and soft tissue infections (SSTI), pneumonia, acute cholecystitis, endophthalmitis, peritonitis, urinary tract infection, splenic abscess, liver abscess, intracerebral abscess, meningitis, cholangitis, and empyema [4]. There were no history of consumption of seafood or exposure to seawater nor prior history of trauma thus, the exact nature exposure of the patient to *V. cholerae* remains un-known. However, it is well-known that immunocompromised patients are more susceptible to several bacterial, viral and parasitic infections especially diabetic patients because of the compromised vascular supply and neuropathy secondary to diabetes [3].

A retrospective study conducted by *Y. Chen*, *H. Tang*, *C. Chao* and *C. La*, collected data from databank of the bacteriology and laboratories from two hospitals in Taiwan, found that a total number of 83 patients were infected with non-O1 *Vibrio cholera* during the years 2009 to 2014. Their results concluded that the two most common underlying diseases were liver cirrhosis and diabetes mellitus, followed by malignancy. Patients’ mean age was 53.3 years and 53 of them were males. In addition, they found that the most common type of infection was gastroenteritis with a total number of 45 patients (54.2%), followed by biliary tract infection with 12 patients (14.5%) and primary bacteremia 11 patients (13.3%). Other infections that were more infrequent included, peritonitis and skin and soft tissue infection (SSTI) with a percentage of 6% for each, followed by urinary tract infection and pneumonia with a percentage of 3.6% and 2.4%, respectively. Furthermore, the overall mortality rate of patients was 7.2% and it was higher with the presence of primary bacteremia, hemorrhage bullae, acute kidney injury, acute respiratory failure or admission to an ICU [4].

No evidence has indicated the extra-intestinal infections of *V. cholerae* in Saudi Arabia. We only found a surveillance for *Vibrio cholerae* in the Eastern Region of Saudi Arabia which was conducted by S. Bubshait, K. Al-Turki, K. Qadri, M. Fontaine, R. Cameron of two years period from January 1996 to December 1997 to detect and prevent local proliferation of imported cholera. The results showed that out of 157 patients, 113 presented with gastroenteritis (72%), and 28 were found through routine screening (18%). All 113 symptomatic patients had short-term gastrointestinal illnesses of 1 to 3 days of duration. Symptoms included diarrhea that did not require hospitalization or intravenous rehydration, abdominal pain, vomiting, and a low-grade fever [5]. Eventually, no statistical studies in Saudi Arabia has been found to state extra-intestinal infections of *V. cholerae* specifically soft tissue lesion which illustrate how rare it is or that there’s not enough reported cases yet. Thus, this is the first case report of extra-intestinal infections of *V. cholerae* in Saudi Arabia.

Upon review of literature, we only found few cases that have been reported of cellulitis due to *Vibrio cholerae* In addition, a degree of hepatic impairment or immunocompromised state like Diabetes Mellitus, chronic infections, malignancies, and peripheral vascular disease are seen in the majority of non-gastrointestinal *V. cholerae* infections which suggests that *Vibrio cholerae* should be included in the differential diagnosis of bacteremic skin and soft tissue infections in patients with underlying illnesses.

Here is a published article by S. Marakia, A. Christidoua, M. Anastasakib and E. Scoulica who reviewed 47 published cases of *V. cholerae* bacteremic skin and soft tissue infections in University of Crete, Greece from January 1974 to May 2015 of a total number of 48 patients. The result was predominated with male gender of 89.6% and Mean age of 57.8 years. Liver cirrhosis, chronic liver disease and alcohol abuse were common comorbidities followed by Diabetes Miletus, solid tumors, hematological malignancies, chronic steroid use and peripheral vascular disease. Furthermore, the soft tissue lesions most commonly described were localized cellulitis, with or without bullous and hemorrhagic lesions (66.7%), while necrotizing fasciitis was the rarest (29.2%) [6]. The review showed that elderly and males are at high risk to be infected with *V. cholerae.* In addition, localized non-complicated cellulitis was the most common soft tissue lesion.

An increase in antimicrobial resistance to antibiotics by *Vibrio cholerae* has been reported in several epidemic outbreaks. A study done by L. Ghanem and N. Elhadi which was published in 2017 to detect the presences of *Vibrio parahemolytics* from the costal water in the Eastern Province in Saudi Arabia. They collected 375 samples from seawater, and it was tested using traditional microbiological techniques, they found 340 samples that were positive for *V. parahemolyticus*. Samples were also tested for antibiotic sensitivity against 28 agents and majority of samples were highly resistant to carbenicillin (98%), ampicillin (88%), and Cephalothin (76%) [7]. The *Vibrio cholerae* in our case showed antibiotic-resistant to ampicillin this indicates organisms, such as *V. cholerae*, should be under continuous surveillance of antimicrobial resistance profile to manage with proper antibiotics.

## 4 Conclusion

*Vibrio cholerae* should be included in the differential diagnosis of any bacteremic skin and soft tissue infections especially in elderly, comorbid and immune-compromised patients. Well-timed, proper antibiotic and surgical treatments are important in the management of the infection to decrease morbidity and mortality.

## Sources of funding

There are no sources of funding for this research.

## Ethical approval

Not applicable. The study is exempt from ethical approval in our institution.

## Consent

Consent has been obtained from the patient’s family after he died. No identifying details have been used in the article.

## Author’s contribution

Authors H. AlMuqallad, A. Alzarra, H. Alrashed and M. Aladwani contributed to the paper by collecting all important data and information pertaining to the case.

Authors M. Aladwani, A. Hakami contributed to the paper by reviewing all the available literature related to the case.

Authors T. Abdelhafiz, A. Alnimr and A. Alabduljabbar contributed to the paper by reviewing the final manuscript.

## Registration of research studies

Not applicable.

## Guarantor

Moudhi Aladwani, Amani Hakami and Abdulrahamn Alabduljabbar.

## Provenance and peer review

Not commissioned, externally peer-reviewed.

## Declaration of Competing Interest

No potential conflict of interest relevant to this article was reported.
